# Interactive Responses of a Thalamic Neuron to Formalin Induced Lasting Pain in Behaving Mice

**DOI:** 10.1371/journal.pone.0030699

**Published:** 2012-01-23

**Authors:** Yeowool Huh, Rushi Bhatt, DaeHyun Jung, Hee-sup Shin, Jeiwon Cho

**Affiliations:** 1 Center for Neural Science, Korea Institute of Science and Technology, Seoul, Korea; 2 Department of Neuroscience, University of Science & Technology, Daejeon, Korea; 3 Yahoo! Labs, Bangalore, India; Georgia State University, United States of America

## Abstract

Thalamocortical (TC) neurons are known to relay incoming sensory information to the cortex via firing in tonic or burst mode. However, it is still unclear how respective firing modes of a single thalamic relay neuron contribute to pain perception under consciousness. Some studies report that bursting could increase pain in hyperalgesic conditions while others suggest the contrary. However, since previous studies were done under either neuropathic pain conditions or often under anesthesia, the mechanism of thalamic pain modulation under awake conditions is not well understood. We therefore characterized the thalamic firing patterns of behaving mice in response to nociceptive pain induced by inflammation. Our results demonstrated that nociceptive pain responses were positively correlated with tonic firing and negatively correlated with burst firing of individual TC neurons. Furthermore, burst properties such as intra-burst-interval (IntraBI) also turned out to be reliably correlated with the changes of nociceptive pain responses. In addition, brain stimulation experiments revealed that only bursts with specific bursting patterns could significantly abolish behavioral nociceptive responses. The results indicate that specific patterns of bursting activity in thalamocortical relay neurons play a critical role in controlling long-lasting inflammatory pain in awake and behaving mice.

## Introduction

Thalamic relay neurons are known to relay peripheral signals to the cortex, except for olfaction [1]. Slice physiological studies have suggested that the reticular thalamus (RT), the main GABAergic input to the thalamus, could enable a single thalamocortical (TC) neuron to switch from tonic firing to burst firing via the presence of T-type Ca^2+^ channels [2]–[6]. This *in-vitro* characteristic of TC neurons to switch between the two firing modes has been suggested to modulate sensory information relayed to the neocortex [7], [8].

Tonic and burst firings have been suggested to serve differential roles. Tonic firing was considered to faithfully relay peripheral sensory signals to the cortex during the awake and vigilant states [9], [10] while burst firing was considered to block sensory signal transmission from being relayed to the cortex during certain phases of sleep or deep anesthesia [9], [11], [12]. This was based on the observation that burst firing event was rare during the awake state, but became more prevalent during sleep or deep anesthesia. Although tonic firing predominates over burst firing in the awake state, studies done in the awake state proposed that burst firing mode could also have meaningful roles such as new stimulus detection in the visual system [13] and whiskering behavior of mice [14].

Burst firing has been implicated to serve different roles from that of tonic firing in many sensory systems [15]. Likewise, the presence of T-type Ca^2+^ channels in lamina Ι spinal cord neurons was shown to aid the development of hyperalgesia by facilitating long term potentiation (LTP) between the C-fiber and the spinal cord projection neuron [16].

However, how the respective TC firing modes encode pain sensation is still elusive [17], and the role of burst firing in pain modulation has been especially controversial, particularly in the awake condition. Ever since abnormally high levels of bursting have been recorded in the somatosensory thalamus of awake patients suffering from central pain syndrome (CPS) [18], such bursting activity has been consistently suggested to be a pathological firing mode that intensify pain in pain patients [19]–[22] and animal models of CPS [23], [24]. However, another clinical study reported that no difference in the frequency of bursting activity existed in the somatosensory thalamus between patients with intolerable pain and patients with motor deficits [25], challenging the idea that increased thalamic bursting could cause pain. A similar result was reported more recently in a rat model of CPS [26]. Further challenging the theory of bursting as a pain carrying signal, α1G knockout mice, lacking low threshold burst spikes (LTS) in the somatosensory thalamus under anesthesia, exhibited a greater visceral pain response than the wild-type littermates in the behavioral assessment [27], implying that bursting may actually act as a blocker of nociceptive information. Due to these controversial reports, the role of burst firing in pain modulation in non-neuropathic and conscious conditions remains unresolved.

Previous studies so far have been carried out in neuropathic pain patients and investigated under anesthesia in animal studies. However, differential involvement of tonic and burst firings in pain signaling of behaving non-neuropathic subjects is poorly investigated. The fact that inconsistent reports on the possible role of burst firing in pain could be due to differences in physiological states only reiterates the importance of understanding pain mechanisms in the awake state of non-neuropathic organisms.

In addition, since TC neurons are prone to bursting during sleep or anesthesia [9], [11], [12], studying pain transmission in the awake state should be more valuable [28]. Use of anesthetics could complicate the interpretation of the role of thalamic bursting in pain. For example, barbiturates, often used anesthetics, are known to potentiate GABA receptors [29]. Since burst firing in the TC is induced by GABAergic input from the RT, studies done under barbiturate anesthesia are likely to exaggerate the effect of burst firing that might lead to misinterpret the role of burst firing in pain. Urethane, another anesthetic, also is known to act on GABA, NMDA, glycine, and AMPA receptors at 100 mM concentrations, which is an often used anesthetic concentration [30]. Theoretically both the excitatory (NMDA and AMPA) and the inhibitory (GABA and glycine) channels could be simultaneously activated by urethane, but urethane effect *in-vivo* has not been well elucidated, making the prediction on the effect of urethane more complicated.

We therefore sought to reliably determine the roles of tonic and burst firings in the ventrobasal (VB) thalami including the ventro-posterior lateral (VPL) and ventro-posterior medial (VPM) nuclei, the homologous structure to the human somatosensory thalamus, during the formalin-induced inflammatory nociception in mice. The formalin test was used as our pain model not only because lasting inflammatory pain induced by formalin is considered an appropriate model of clinical pain [31], but also because it provides a better paradigm for comparing neural responses before and after the pain induction. It is a well studied pain model which exhibits the characteristic 1^st^ and 2^nd^ phase behavioral nociceptive responses separated by the interphase. The 1^st^ phase is considered to be due to direct stimulation of nociceptors, the 2^nd^ phase is considered to be due to inflammation that develops in response to formalin [32], and the interphase is suggested to result from active inhibition from the periphery [33].

Using the single unit recording technique, we recorded and intensively analyzed changes in firing patterns of VB neurons during the course of lasting nociceptive pain induced by formalin. Furthermore, electrical stimulations mimicking certain properties of thalamic bursts were given to verify the significance of bursting properties in pain modulation. We report that both tonic and burst firing modes are involved in encoding nociceptive pain and that bursts with specific properties have an anti-nociceptive effect.

## Results

### Behavioral and Thalamic Responses to Formalin

**Figure 1:**
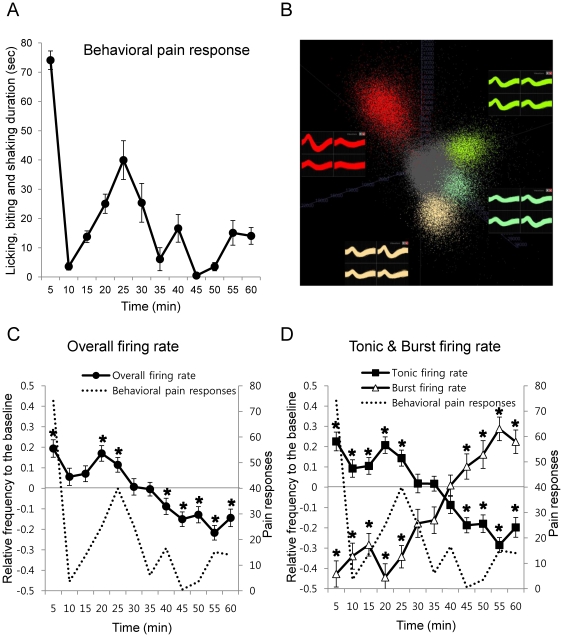
Behavioral nociceptive responses and temporal changes in VB neuronal firing patterns induced by formalin in behaving mice. (A) Behavioral pain responses to formalin analyzed in 5 min segments (F = 14.42, p<0.01). All data points are mean±SEM. n = 9 mice. ANOVA with Repeated measures were used for statistical analysis over time. (B) Spike sorting sample from a tetrode. (C) Normalized overall VB neuronal firing rate changes to formalin over time in 5 min segments. (D) Normalized tonic firing and burst firing rate changes to formalin over time in 5 min segments. (C and D) n = 48 neurons, 7 mice. All data points are mean±SEM. Dotted line is the behavioral nociceptive responses superimposed for comparison with the VB neuronal firing responses. Student's t-test was used to compare each data points with the baseline. *indicates significant differences at p<0.05.

To investigate the differential role of tonic and burst firings in pain modulation, single VB neuronal activities to nociceptive stimulus (5% formalin) was measured in behaving mice. Behavioral responses and VB neuronal activities were measured in separate sets of experiments because the recording cable and implanted microdrive interfered with the expression of certain nociception related behaviors such as licking and biting. Meanwhile general movements were not hindered by the cable or the microdrive. Subcutaneous injection of formalin to the footpad of the hind limb induced the acute 1^st^ phase and lasting 2^nd^ phase behavioral responses which were separated by the quiescent interphase (Figure 1A).

**Figure 2:**
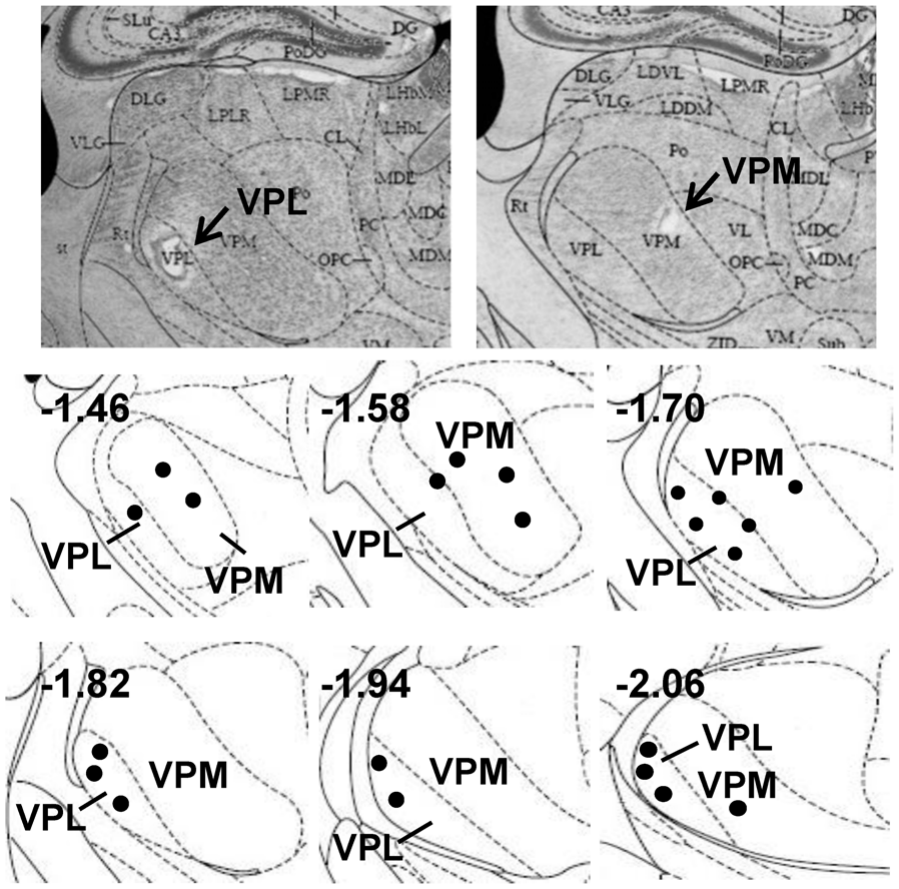
Histology and schematic drawing indicating all recording locations. Numbers on the left corner of each drawing indicates millimeter distance from the bregma.

In a separate experiment, VB thalamic responses to formalin-induced nociception were recorded in behaving mice. Samples of spike-sorted single units from a single tetrode are shown in Figure 1B. The recording locations were verified by histological examinations and marked in Figure 2. Since baseline firing rates varied between neurons, individual responses of 48 single neuron activities were normalized in order to reveal relative changes to the baseline (see methods).

Temporal fluctuations in the overall firing rate strikingly mirrored the phasic changes in the behavioral responses (Figure 1C). For example, overall firing rate increased significantly relative to the baseline firing rate during the time course corresponding to high levels of behavioral responses during the 1^st^ and 2^nd^ phases (0–5 min and 15–25 min, respectively), while it decreased significantly relative to the baseline firing rate during the time course corresponding to low behavioral pain responses (35–60 min).

Since the capability of thalamic neurons to switch between tonic and burst firing has been suggested to play different roles in sensory signal modulation by previous slice physiological studies [7], [8], the contributions of each firing mode in nociceptive signal encoding were investigated by identifying tonic and burst firings from individual spike trains. The definition of LTS burst (≥100 ms of preceding silent period and ≤4 ms of inter-spike-interval [34], [35]) was used to separate burst spikes from tonic spikes because most LTS bursts recorded *in-vivo* met these criteria. Tonic and burst firing rates (not normalized, Hz) at different phases of the behavioral response are summarized in Table 1. Interestingly, tonic firing was predominant over burst firing at all times, both before and after pain induction, which is consistent with studies reporting that tonic firing is the predominant firing mode in the awake state [9], [10]. Nevertheless, formalin injection resulted in dynamic changes in both tonic and burst firing rates. For example, average tonic firing rate significantly increased by approximately 2 Hz during the 1^st^ high pain phase (0–10 min, p<0.05) and significantly decreased by approximately 1.5 Hz during the late 2^nd^ low pain phase (35–60 min, p<0.05) compared to the baseline (−10∼0 min) of tonic firing rate (Table 1). On the contrary, burst firing rate decreased significantly during the 1^st^ and early 2^nd^ phases (0–35 min, p<0.001), while it increased significantly during the late 2^nd^ low nociceptive response phase (35–60 min, p<0.05). These results clearly indicate that both tonic and burst firings are actively involved in modulating nociceptive signals in the awake state.

**Table 1:** Responses of VB Neurons to Formalin Induced Pain before Normalization.

		Tonic	Burst Spike
**Baseline**	**FR (Hz)**	6.53±0.75	0.38±0.06
**(−10∼0 min)**	**ISI (s)**	0.35±0.05	20.63±4.00
	**ratio (%)**	94	6
**1st phase**	**FR (Hz)**	8.60±0.57	0.10±0.01
**(0–10 min)**	**ISI (s)**	0.22±0.02	33.23±4.35
	**ratio (%)**	99	1
**Early 2nd**	**FR (Hz)**	7.76±0.35	0.19±0.02
**phase**	**ISI (s)**	0.28±0.03	30.03±2.57
**(10–35 min)**	**ratio (%)**	98	2
**Late 2nd**	**FR (Hz)**	4.88±0.31	0.62±0.05
**phase**	**ISI (s)**	0.59±0.06	13.00±1.27
**(35–60 min)**	**ratio (%)**	89	11

Baseline is the spontaneous neural activity before formalin injection. Neural response after formalin injection is divided into the 1^st^ and 2^nd^ phases based on the quiescent interval between the two peaks of the behavioral pain responses. Early and late phase division in the 2^nd^ phase is also based on the same rationale. n = 48 neurons, 7 mice. All values are mean±SEM. FR: firing rate, ISI: inter-spike-interval, ratio: percentage of respective firing modes from the total number of spikes.

In order to figure out more detailed patterns of the changes in both tonic and burst firings in relation to the changes in the behavioral nociceptive responses, we analyzed the changes of tonic and burst firing rates of individual neurons relative to the baseline in 5 min segments (see methods). The averages of normalized tonic and burst firing rates of each time segment across all neurons revealed distinct and detailed relationships between both firing modes and the behavioral nociceptive responses. For example, the timings of biphasic change in tonic firing rate were almost identical to those of the behavioral responses in the time domain (Figure 1D, Tonic firing rate), supporting the idea that tonic firing reflects peripheral nociceptive activity [28], [36]. Accordingly, tonic firing rate was positively correlated with the behavioral responses over time (Pearson correlation coefficient = +0.686, p<0.05). Contrary to the response of tonic firing, burst firing rate was negatively correlated with the behavioral responses over time (Pearson correlation coefficient = −0.607, p<0.05). Interestingly, burst firing rate was initially suppressed below the baseline but gradually started to increase right before the 2^nd^ phase behavioral responses declined (15–20 min after formalin) and remained above the baseline after 45 min (Figure 1D, Burst firing rate). This indicates that burst firing has a tendency to be suppressed during the initial phase of lasting nociceptive pain, but becomes potentiated only after prolonged nociceptive pain in conscious conditions. Since bursting of VB neuron in mice is controlled mainly by the RT input [6], suppression of burst firing during the initial phase of nociception would be due to the reduced input from the RT while potentiation of burst firing would be due to relative increase of the RT input to the VB, even though the mechanism of when and how the RT would be activated to the lasting pain signal is not known at the moment. Overall, the temporal patterns of tonic and burst firing rates that are strikingly correlated with those of behavioral pain responses suggest that the dual firing modes of VB neurons are differentially coordinated in concert to code for nociceptive pain information in the awake state.

To investigate whether there are different neuronal response types in the VB, we also examined temporal changes in individual neurons' activities and found that all cells were responsive to nociceptive stimuli despite the variations existing across individual neurons. The dominant pattern of tonic firing rate change included a biphasic increase corresponding to the biphasic increase in behavioral responses (85%, 41 out of 48 cells) while the dominant pattern of burst firing rate change was the sustained increase starting from the time of behavioral 2^nd^ phase nociceptive pain reduction (92%, 44 out of 48 cells), similar to the trend shown in Figure 1D. Minor deviations of tonic firing rate change patterns (15%, 7 cells) were persistent increase or decrease of firing rate that had no apparent temporal correlation with behavioral responses. The only deviation in the burst firing rate change pattern (8%, 4 cells) was the biphasic increase corresponding to the biphasic increase in behavioral responses, similar to the stereotypic tonic firing pattern. This indicates that most of the recorded cells responded in a stereotypical pattern.

### Interaction between Tonic and Burst Firing

Since changes in tonic and burst firing rates slightly preceded the 2^nd^ phase of the behavioral response to the formalin injection, either the decreased tonic firing or increased burst firing could have led to the change in behavioral nociceptive responses. In order to investigate the interactive relationship between burst and tonic firing, a cross-correlation analysis between tonic and burst spikes was performed using tonic spikes as reference. Results revealed that the timing of the rise in burst firing preceded the timing of the fall in tonic firing by 8 ms, implicating the possibility that burst firing acts as a trigger to attenuate tonic firing in VB neurons. These observations suggest that the decrease of tonic firing rate shown 35 min after the formalin injection could possibly be induced by thalamic bursting and not solely by the reduction of incoming nociceptive signals from the spinal cord, because prolonged increase of firing (up to 90 min) in the spinal cord of anesthetized rats in response to formalin has been previously reported [33]. Subsequently, relative decrease in tonic firing by increased burst firing of individual neurons could have reduced behavioral nociceptive responses.

### Burst Properties and Behavioral Nociceptive Responses

**Figure 3:**
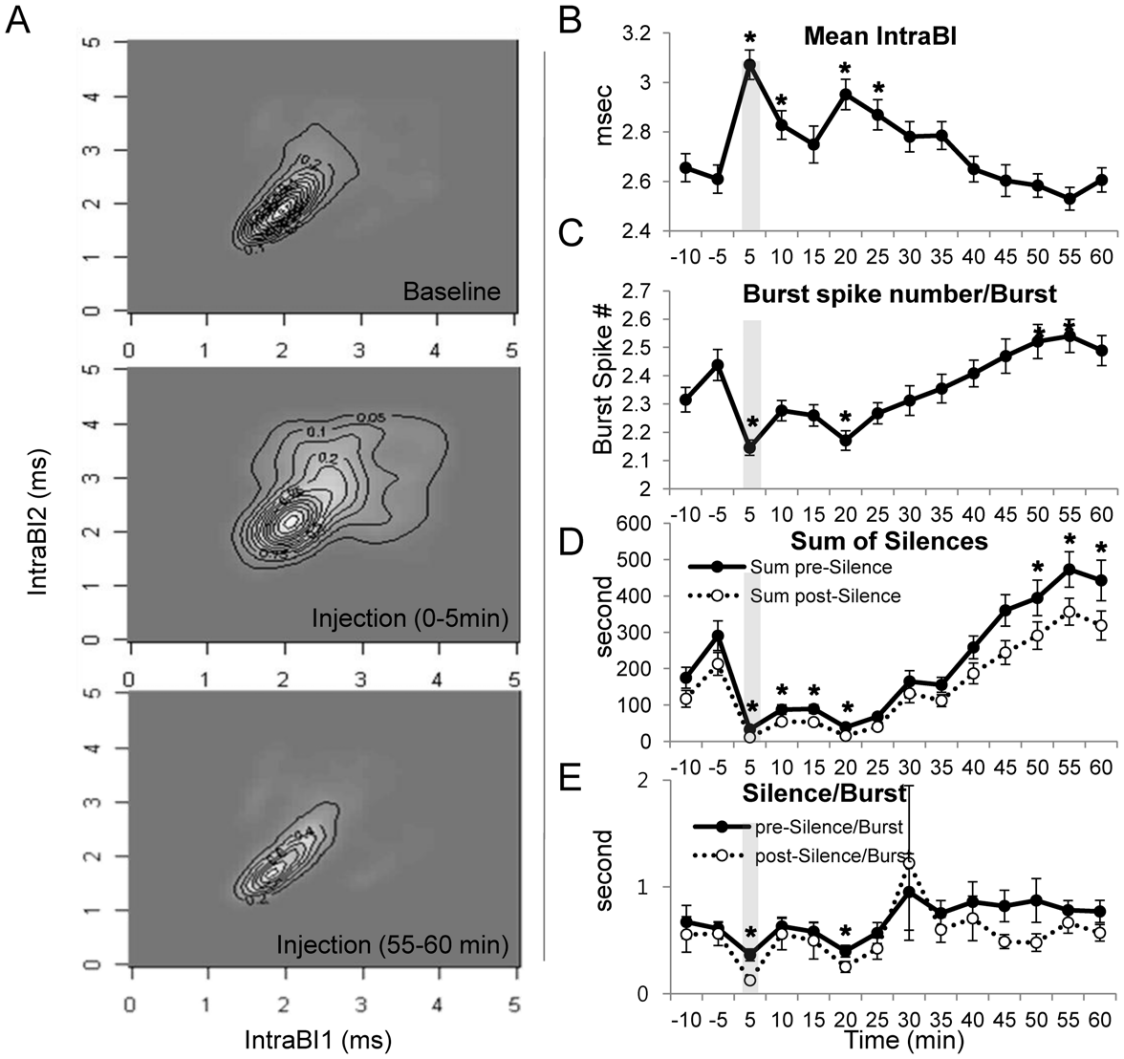
Temporal changes of burst properties before and after formalin injection. (A) Contour maps for JPD of the 1^st^ and the 2^nd^ IntraBI for baseline and different pain response phases after formalin injection. (B) Mean of all IntraBIs to formalin over time. (C) Number of burst spikes within a burst changes to formalin over time. (D) Sum of pre- or post-silent periods per cell changes to formalin over time. (E) Pre- or post-silence per burst changes to formalin over time. (B–E) Vertical grey stripes indicate the formalin injection point. All data points are mean±SEM. To compare each data point with the baseline, student's t-test was used. * indicates significant difference at p<0.05. n = 48 neurons, 7 mice.

Acknowledging the potential importance of burst firing in nociceptive pain signaling, we investigated whether any changes in bursting properties correlated to the changes in behavioral nociceptive responses. Since VB neuronal responses mirrored the time course of behavioral nociceptive responses, which was measured separately, we assumed that bursting property changes would also correspond to the behavioral responses. Interestingly, we found that burst properties changed in parallel with the changes in the behavioral nociceptive responses. For example, contour maps of joint probability density (JPD) between the consecutive pairs of the 1–4^th^ intervals of burst spikes within a burst (IntraBI1, 2, 3, and 4, respectively) displayed widened IntraBIs when temporally corresponding behavioral nociceptive responses peaked, while also displaying tightened IntraBIs as behavioral nociceptive responses diminished. Figure 3A qualitatively illustrates how IntraBI1 and IntraBI2 systemically change in response to nociception over time. The remaining consecutive pairs (IntraBI2∼3 and 3∼4) were not shown because their patterns were identical with those of the first pair (IntraBI1∼2). Consistently, changes in mean IntraBI of all IntraBIs quantitatively showed this trend (Figure 3B). Mean IntraBI significantly increased compared to that of the baseline at time intervals corresponding to high behavioral nociceptive responses (0–10 min and 15–25 min after formalin), while spike number per burst was inversely correlated with the behavioral nociceptive responses (Figure 3C). The nature of the relationship between burst spike number and the first IntraBI was inversely proportional (Pearson correlation coefficient = −0.642, p<0.01), meaning that bursts with shorter IntraBI1 had a tendency to have more burst spikes than bursts with a longer IntraBI1.

In addition, we also analyzed the sum of silent periods immediately before and after a burst (peri-burst-silences) as a rough estimation of total neuronal suppression for individual VB neurons, because characteristic hyperpolarizations before and after LTS are important components of a rebound burst that contributes to neuronal suppression in the thalamus [4] [37]. The sum of peri-burst-silences were calculated by adding all the inter-spike-interval lengths that occured before and after a burst in 5 min time segments of individual cells and then the average of all recorded cells was plotted. The sum of peri-burst-silences increased significantly as the behavioral nociceptive responses diminished (Figure 3D), supporting the idea that suppressed tonic activity is due to thalamic hyperpolarizations accompanying LTS bursts. Furthermore, increased summation of peri-burst neural suppressions is likely a consequence of increased occurrence of LTS since peri-burst-silence per burst is nearly constant over time once bursts start to be potentiated (Figure 3E).

### Importance of Bursting Property for Relieving Nociceptive responses in Brain Stimulation

**Figure 4:**
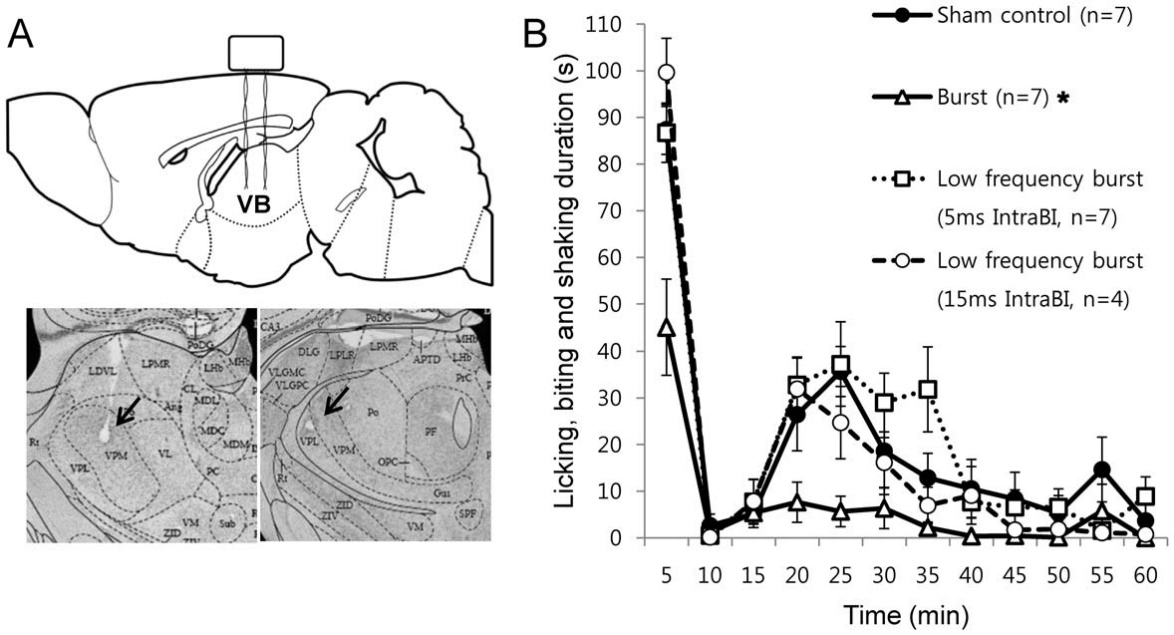
Alterations in pain responses by different electrical stimulation conditions during the formalin test. (A) Schematic drawing and histology sample of stimulation sites. (B) Behavioral pain responses to burst (3 ms IntraBI) or low frequency burst (5 ms and 15 ms IntraBI) stimulations compared with the sham control. All stimulation conditions were composed of 5 burst spikes and the total stimulation frequency was set to be ∼8 Hz by modifying inter-burst-intervals. Kolmogorov-Smirnov Z test showed that only burst stimulation condition significantly reduced pain responses compared to that of the sham control. (* p<0.01).

To demonstrate that burst properties are critical for relieving nociceptive pain responses, we compared formalin induced behavioral nociceptive responses of mice under 2 different VB electrical stimulation conditions, burst (3 ms IntraBI) or low frequency burst (5 ms IntraBI), with the sham control. Schematic alignment of the stimulating electrodes and a sample of the stimulation sites are depicted in Figure 4A. The burst stimulation condition (3 ms) was chosen to be within the definition of a LTS burst used in the single unit recording analysis, while the low frequency burst stimulation condition (5 ms) was designed to slightly deviate from our burst criterion in terms of IntraBI length. Besides the 1 ms deviation from our burst definition, the low frequency burst stimulation condition was set to have equal stimulating conditions as the burst stimulation condition. Both groups received stimulations consisting of a series of bursts in which each burst had 5 bipolar square pulses (100 µA) with a 600 ms interval that separated the bursts for the entire experimental period. None of the stimulation conditions caused any visible irritation or discomfort in mice. Results showed that the burst stimulation (3 ms IntraBI) effectively and significantly diminished behavioral nociceptive responses compared to those of the sham control, while the low frequency burst stimulation (5 ms IntraBI) had no such effect even though both stimulation conditions had a similar total stimulation frequencies (approximately 8 Hz, Figure 4B). This result showed that the ability to reduce the behavioral nociceptive responses appears to critically depend on the property of stimulation, IntraBI in this case, because a slight increase in IntraBI by only 2 ms abolished the nociceptive pain reduction ability of burst stimulation in the low frequency burst stimulation condition. This indicates that precise bursting properties, especially the IntraBI, and not the total frequency, are required for effective control of nociceptive pain.

Additional two preliminary tests, 2 ms and 15 ms IntraBI stimulations, were carried out to respectively investigate whether shorter IntraBI interval or periodicity was important in yielding an anti-nociceptive effect of burst stimulations. The effect of 2 ms stimulation was inconclusive due to intermittent seizures. The 15 ms stimulation induced no aberrant behavior, but had no effect in reducing nociceptive responses compared to the sham control (Figure 4B).

## Discussion

The present study demonstrated that the change of behavioral nociceptive pain responses was reliably represented by temporally corresponding changes in both tonic and burst firings of VB neurons in behaving mice. In the awake state, the overall firing rate as well as the tonic firing rate of VB neurons reliably reflected the behavioral nociceptive responses while the burst firing rate soundly represented the decreased behavioral nociceptive responses.

Our finding on burst firing may offer a clue to resolve the present controversies on the role of burst firing in nociceptive pain. As mentioned in the introduction, the role of VB neuronal burst firing in pain has been especially controversial. Some studies reported that increased bursting is associated with increased pain in neuropathic patients or animals [19]–[22], [24] while other studies reported that there is no significant correlation [25], [26]. Yet, another study using genetically mutated mice and anesthetized recordings suggested that the absence or excess of burst firing could be correlated with the increase or decrease of nociceptive pain responses, respectively [27], [38]. It is noteworthy to mention that discrepant findings on the role of burst firing in all previous studies could be due to a couple of factors. First and foremost, different physiological conditions used in different studies could have been the biggest factor of controversy. For example, studies reporting increased bursting occurrence in association with greater pain perception were done under neuropathic pain conditions [18]–[20], [22], [24], in which burst firing properties might have been altered. Indeed, a study that investigated the changes in burst firing property after spinal cord injury leading to neuropathic pain reported that burst firing properties, such as burst length, silences, and IntraBI were different in the VPL thalamus compared to those of the sham surgery group [24]. Furthermore, anesthetics used during the recordings of neural activity must also have influenced the interpretation of the results because most animal studies were carried out under anesthesia. Studies done under anesthesia failed to show temporal correlation between fluctuations of burst firing pattern and changes of nociceptive pain responses [27], [38]. Anesthesia is known to depress cortical neuronal activity [39], and reduction of corticothalamic input was shown to decrease VB neuronal activity [40]. Therefore, suppression of cortical activity would reduce cortical influence on the TC relay neurons or the RT and might alter its influences on TC neuronal activities, modulating TC neurons to respond differently to nociceptive pain stimuli under anesthesia. Therefore, pain studies done under anesthesia might be insufficient to explain the pain-signaling mechanism of the awake state. Taken together, pain with different pathological and physiological conditions could have different mechanisms in terms of thalamic pain modulation.

Another intriguing finding is that not only the presence of bursts, but also the properties of bursts could be important in reducing nociceptive pain responses. During recordings, IntraBI, the number of burst spikes and peri-burst silences systemically changed in accordance to the changes in the behavioral nociceptive pain responses. Among these properties, IntraBI was demonstrated to be an important component in reducing behavioral nociceptive pain responses with electrical VB stimulation: burst stimulation (3 ms IntraBI) effectively reduced nociceptive responses while low frequency burst stimulation (5 ms IntraBI) had no such effect. Additional stimulation studies were carried out to delve whether shorter IntraBI, rhythmicity, or possibly both are accountable for the anti-nociceptive effect by stimulating with 2 ms or 15 ms IntraBI. Seizures induced in 2 ms IntraBI stimulation made it difficult to reliably measure its effects. Artificial stimulations overriding the naturally occurring signals may be the cause of seizure induction. However, this is not an indication that naturally occurring bursts with shorter IntraBI would be ineffective. Since the single unit recording results showed that IntraBI tended to decrease up to approximately 2 ms during the time segment corresponding to reduced behavioral nociceptive responses, bursts with shorter IntraBI may be more effective in reducing nociceptive responses in natural conditions. The 15 ms IntraBI stimulation was chosen to make it a multiple of both 3 and 5 ms IntraBI to test the role of rhythmicity in producing anti-nociceptive effect, but it was ineffective in reducing nociceptive behavior. However, this result is not conclusive since the test was done with a small sample size (n = 4). Further studies testing various stimulation conditions with greater sample sizes should be necessary to figure out the bursting parameters for anti-nociceptive effect.

Nonetheless, our neural recording results clearly demonstrate that property changes in burst firing are closely correlated with those in behavioral nociceptive pain responses. This indicates that bursting properties—such as IntraBI, burst spikes number per burst, and silences—may modulate the degree of nociceptive pain transmitted to the cortex. It is interesting to note that these properties corresponding to nociceptive pain relief should more potently activate postsynaptic neurons. As mentioned earlier, short IntraBI and a greater number of burst spikes were suggested to allow greater temporal integration of signals and ensure reliable signal transmission [41]. Silences immediately preceding spontaneous firings of TC neurons were also suggested to facilitate the activation of cortical neurons in restrained awake rabbits [42]. Taken together, this suggests that there may be an additional nociceptive pain inhibitory system at the cortical level that is modulated by thalamic bursting activity via specific inhibitory cortical neurons.

The exact mechanism of how specific bursting could lead to reduction of nociceptive pain behavior is unclear, but we can assume that it occurs possibly through the mutual and complicated interactions of the RT and cortex with the VB. *In-vitro* experiments have shown that VB neurons are able to fire in LTS bursts via the presence of T-type Ca^2+^ channels which could be activated only after >100 ms of hyperpolarization [35]. Since the RT is the major GABAergic source to VB neurons in rodents [43], RT activation should depress the activity of VB neurons and then induce LTS bursts in the VB. In turn, the LTS bursts in the VB could potentiate the RT again to generate more LTS bursts in the VB. The regenerative LTS burst production, accompanied by hyperpolarization, could lead to greater depression of VB neurons which blocks nociceptive pain signals at the thalamic level. In addition, potentiated post-synaptic responses presumed by increased thalamic burst activity suggests that nociceptive pain reduction at the cortical level by VB neuronal burst firing may be mediated by cortical inhibitory neurons rather than excitatory ones. This assumption is supported by previous studies showing that burst spikes were shown to more potently activate cortical neurons than tonic spikes [42], [44] and that TC neurons were suggested to have more potency to activate the cortical inhibitory interneurons than the excitatory ones [45]–[47]. Taken together, it is likely that increasing inhibition in the cortex by VB neuronal burst firing might block the nociceptive pain transmission at the cortical level in addition to thalamic blocking of nociceptive pain signals.

Although the ability of burst firings in reducing nociceptive responses have been demonstrated, our electrical stimulation study could not demonstrate that tonic firing faithfully transmits nociceptive pain signals despite the fact that tonic firing frequency mirrored the changes in behavioral nociceptive pain responses. Our attempt to amplify behavioral nociceptive responses using electrical stimulation in tonic modes with several frequencies following the formalin injection failed to increase stereotypic behavioral nociceptive pain responses (data not shown). However, this does not preclude the possibility that tonic firing could be a pain carrying signal. Thalamic relay of nociceptive pain signals to the cortex may require more than occurrence of tonic firing in the VB because an increase of only 2 Hz in tonic firing shown in neural recordings was strongly correlated with an increase of behavioral nociceptive pain responses. Failure to augment nociceptive pain responses using tonic stimulation suggests that reliable nociceptive pain transmission requires thalamic tonic firing to be resonated with the incoming pain signals from the spinal cord, co-activation of other brain areas for successful isolation or identification of the nociceptive pain information from other modalities of sensory signals. Interestingly, during electrical stimulation in tonic mode, animals showed increased grooming behaviors other than stereotypic pain responses, suggesting that tonic stimulation might have amplified other sensations as well, which, in turn, might have hindered the expression of nociceptive pain responses. Since the VB neurons relay many sensory modalities other than pain, other sensory signals such as touch [48] would be intermingled with the nociceptive pain signals. Exactly how pain signals are distinguished from other sensory signals is uncertain at the moment and the role of tonic firing in nociceptive pain transmission cannot be precisely determined.

Meanwhile, the increased tonic firing during the VB neuronal recordings could have been due to mainly excitatory inputs from the brainstem or the cortex. Firing mode change from a single VB neuron was shown to be controlled by the inactivation and activation dynamics of T-type Ca^2+^ channels, which de-inactivates after >100 ms inhibitory input [35]. Therefore, the firing mode of thalamic neurons would depend on the balance of excitatory and inhibitory inputs. Since the corticothalamic connection is excitatory [49] and uses glutamate as a neurotransmitter [50], direct cortical input to the VB could have promoted VB neurons to fire in tonic mode. However, due to the complexity that the cortical inputs also innervate the RT [1], which is the main inhibitory source for VB neurons, cortical activity could also promote VB neuronal burst firing by activating the RT more than the VB. Like the cortical input, influence from the brainstem on the VB could also be complex [51]–[54].

In our experiment, electrical stimulation with a specific IntraBI effectively reduced nociceptive pain responses. Whether it also blocks other sensory signals such as touch or temperature, which are also relayed in the VB, could not be tested, but is possible since paraesthesia is the most common side effect of patients with deep brain stimulation (DBS) therapy for chronic intolerable pain. However, paresthesia and other side effects [55]–[57] could be due to continuous high frequency stimulation. Many DBS stimulation protocols for pain relief employed continuous high frequency stimulations, mostly >100 Hz [58]–[60]. The efficacy of the high frequency stimulation for pain control was also questionable since high frequency stimulation efficacy varied between individuals [58], [59], and most thalamic stimulation produced long term pain relief in only approximately 50% of patients experiencing neuropathic pain [61]. By understanding firing properties related to pain relief, including those of bursts, DBS stimulation protocols for pain relief could become more effective. However, since anatomical distinctions exist between human and rodent thalami, understanding the firing properties of the human pain related thalamic nuclei such as the ventrocaudal (VC) or posterior part of the ventral medial nucleus (VMpo) would provide better stimulation strategies in a clinical setting.

The mechanisms on how high frequency DBS used in therapy exerts its therapeutic effect—by activation or inhibition—are still elusive [62]. Basically DBS effect would occur by electrical stimulation of neural elements [56]. *In-vitro* slice studies showed that application of high frequency stimulation has an inhibitory effect [63]. However, anti-nociceptive effect by electrical stimulation shown in our study may have occurred through a different mechanism, since we used intermittent burst stimulation with very low total stimulation frequency (∼8 Hz) whereas the *in-vitro* slice studies used continuous high frequency stimulation.

Another stimulation method used for therapeutics is the transcranial magnetic stimulation (TMS) [64]. A study stimulating the cortex with different theta burst stimulation (TBS) protocols using TMS demonstrated that the different TBS protocols had different action mechanisms on the cortex, for example, intermittent TBS increased cortical excitability while continuous TBS depressed cortical activity [65]. Although continuous TBS was shown to effectively reduce acute pain perception [66], [67], its effect on longer lasting or intractable pain has not yet been demonstrated, but may also be effective on those conditions since continuous TBS depresses cortical activity.

In addition to the VB, the thalamic nucleus submedius (Sm) is another thalamic nucleus implicated to have importance for nociceptive pain modulation in animals. Behavioral studies showed that electrical or chemical activation of Sm exerted anti-nociceptive effect possibly through the Sm-ventrolateral orbital cortex (VLO)-periaqueductal grey (PAG) connection, which is involved in the descending pain control [68]–[70]. Although our DBS stimulation could have also activated the Sm, the anti-nociceptive effect of the burst stimulation is unlikely to have occurred by Sm activation alone, since the low frequency burst stimulation (5 ms IntraBI) had no such effect.

Our results provide a clear reference regarding the role of TC dual firing modes in freely behaving mice in order to offer a better understanding of nociceptive pain modulating mechanisms in the TC circuit of behaving mice. The standard reference from awake wild-type mice should be particularly important for understanding genetic and molecular mechanisms of thalamic nociceptive modulation because the variability of genetic backgrounds can possibly affect the characteristics of wild type littermates of mutants [71].

In summary, both tonic and burst firings in the VB were shown to be intricately coordinated to orchestrate behavioral nociceptive responses in awake and freely moving mice. In addition, the role of specific bursting in anti-nociceptive effect was successfully demonstrated using electrical brain stimulation. More importantly, our data suggest that the properties of thalamic bursting such as IntraBI are critical in modulating inflammation mediated nociceptive pain signal transmission.

## Materials and Methods

### Ethics Statement

Animal use procedures were in accordance with the guidelines of the Institutional Animal Care and Use Committee of Korea Institute of Science and Technology.

### Subjects

First generation male mice of C57BL/6J×129/SvJae hybrids were used in the experiment. Mice were maintained with free access to food and water under a 12∶12 hour light∶ dark cycle, with the light cycle beginning at 8:00 AM. Prior to all tests, mice were handled for a week and habituated to the experimental setting for at least 20 minutes.

### Behavioral nociception assessment

The formalin test was used for the behavioral nociceptive pain assessment. Nociception was induced by injecting 10 µl of 5% formalin (1∶20 dilution of 37% formalin solution in double deionized H_2_O) to the left footpad of each mouse (n = 9, 10–12weeks, body weight 22–28 g). Immediately after the injection, behavioral nociceptive responses were videotaped for an hour. The results were analyzed by at least two blinded investigators and averaged. Nociceptive pain responses were scored by measuring the licking, biting, and shaking duration of the formalin injected paw.

### Microdrive implant surgery for extracellular single unit recording

Mice (n = 7, 10–14weeks) were anesthetized with zoletil (30 mg/kg i.p.), and supplementary doses, one third of the first injection, were given to maintain sufficient levels of anesthesia throughout the surgery. Anesthetized mice were fixed onto a stereotaxic instrument (David Kopf Instruments, USA) for surgery. After drilling a hole above the VB (VPL and VPM), a microdrive with four tetrodes (four 12.5 µm nichrome aromatic polyimide-insulated microwires intertwined into a tetrode, Kanthal precision technology, Sweden; recording tips of each microwire were gold plated to 400–500 kΩ) was placed into the right VB region (AP: −1.58, ML: −1.8, DV: −3.25) and secured onto the skull with stainless steel screws and dental cement. Mice were allowed to fully recover from surgery for a week before recording sessions started.

### Extracellular single unit recording

Recordings were done in a dark room with a white noise generator operating at a maximum of 85 dB. Mice were allowed to habituate in the recording chamber for at least 20 min. Data were obtained with a data acquisition system (Cheetah, Neuralynx, USA). Signals were amplified with gains of 5000–20,000, filtered with a digital signal processing filter at low cut 0.6 kHz and high cut 6 kHz, and sampled at 30,303 Hz. Time stamps and waveforms of neural signals were directly recorded to the PC via the Cheetah data acquisition software. Once single unit signals were successfully isolated, experimental sessions began. Spontaneous neuronal firing of each mouse was recorded for 10 min as a baseline. To the left hind footpad 10 µl of the 5% formalin solution was injected subcutaneously and neural activity was recorded for 60 min after the formalin injection.

### Extracellular single unit recording data analysis

Only well-isolated single units confirmed to be in the VB (VPM and VPL) by histology were used in the analysis (48 neurons from 7 mice). Data obtained via Cheetah data acquisition software were cluster-cut into single units with the SpikeSort3D (Neuralynx, USA). Each cluster-cut unit was verified as a signal from a single neuron by confirming that no spike counts existed under 1 ms in the inter-spike interval histogram of a single unit. Isolated units were further confirmed that they were single units by cross-correlation.

Spikes within a single unit were analyzed by parameters such as firing rates and burst firing properties. Single unit spikes were differentiated into tonic or burst based on inter-spike-intervals. Burst spikes were defined by spikes consisting of at least 2 spikes occurring ≤4 ms with ≥100 ms preceding silence [35]. All non-burst spikes were considered to be tonic. Then, firing rates (Hz) of overall, tonic, and burst spikes before and after formalin injection were analyzed in 5 min segments. Due to considerable variations in baseline firing rates of each cell, firing rates after formalin injection were normalized to reveal the firing pattern change over time. Normalization was done on individual cell basis and then averaged for all recorded cells. Normalization was done as the following: (firing rate after formalin injection−baseline firing rate)/(firing rate after formalin injection+baseline firing rate). This normalization method gives an accurate representation of the average neural response change relative to the baseline, but does not show the magnitude of change relative to the baseline.

Pearson correlation analysis was performed to reveal the relationship between the normalized tonic and burst firing rates and the normalized behavioral nociceptive responses. Behavioral nociceptive responses were normalized as the following: (pain responses−average of pain responses)/(pain responses+average of pain responses). Behavioral nociceptive responses were the formalin induced nociceptive responses of individual subjects analyzed in 5 min segments. Average of nociceptive responses was the mean nociceptive response duration of all 9 mice analyzed in 5 min segments.

Cross-correlation analysis between tonic and burst firing across all neurons was performed to determine whether burst firing preceded tonic firing. The analysis was carried out for the 15–20 min segment following formalin injection, which is the inflection point of both tonic and burst firing, with 1 ms bin width. Auto-correlation was also performed for tonic and burst firing independently to check if neural firings were influenced by any oscillations.

Joint probability density, IntraBI, burst spike number per burst, sum of peri-burst silences, and pre- and post-silence per burst were used for burst firing property analysis. Contour maps for joint probability densities of the 1^st^ and 2^nd^ IntraBI were computed in consecutive pairs to illustrate the joint probability between an IntraBI and the immediately following IntraBI over time in 10 min segments. Other analyses were done in 5 min segments.

### Electrical stimulation of the ventrobasal thalamus

Mice (10–12 weeks) were chronically implanted with two Teflon-coated stainless steel bipolar stimulating electrodes (0.003″ bare 0.055″ coated, A-M Systems, USA) in the VB (AP: −1.34, ML: −1.8, DV: −3). The two bipolar electrodes, approximately 0.6 mm apart, were implanted to align with the anteroposterior axis of the VB for microstimulation. Mice were then allowed to recover for a week, during which they were handled daily. After recovery, mice were electrically stimulated with pulses differing in IntraBI intervals (2, 3, 5 or 15 ms between burst spikes and modified inter-burst-interval to fix the total stimulation frequency to approximately 8 Hz). All stimulating pulses were biphasic square pulses with current amplitude of 100 µA and duration of 100 µs. The sham control group received the same surgical and experimental procedures without electrical stimulation. Behavioral nociceptive pain responses of different stimulation conditions and the sham control group during the formalin test were measured. Conditions for the formalin test and behavioral scoring were identical as described above in the formalin test section. Nociceptive pain behaviors were analyzed by at least two blinded investigators and results were averaged. Stimulation sites were verified with histology.

### Histology

Locations were verified after completion of recording or stimulation. Mice were overdosed with 2% avertin, and a micro-electrolytic lesion was made by passing current through the recording electrode (5–20 µA, 10 s). No current was passed through the stimulating electrodes because the electrode tracts were thick enough to be visualized under a microscope. Then, mice were perfused transcardially with 10% formalin (1∶10 dilution of 37% formalin solution in 0.9% saline). Brains were removed and fixed in 10% formalin (1∶10 dilution of 37% formalin solution in ddH_2_O) for a day and stored in 30% sucrose solution at room temperature for at least a week before sectioning. Coronal sections (50 µm) were cut through the entire thalamus formation with microtome cryostat (Microm, Germany). The sections were stained with Cresyl Violet (Sigma, USA) and examined under a light microscope to determine recording or stimulation sites.
